# Abl Is Able to Inhibit Cell Engulfment

**DOI:** 10.1371/journal.pbio.1000104

**Published:** 2009-04-28

**Authors:** Richard Robinson

Programmed cell death is essential to development—it's how the tiny mitten of a growing hand becomes a glove of individuated fingers, for instance. To dispose of all those cellular corpses, nearby cells (or roving specialists such as the immune system's macrophages) engulf their dead neighbors and digest the remains. Engulfment, therefore, is a valuable process, and it is known to be promoted by two parallel signaling pathways. But preventing engulfment is also important (one can almost hear a little cell's faint cry, “I'm not dead yet!”); however, little is known about how the process is inhibited. In a new study, Michael Hurwitz and colleagues describe a pathway that does just that.

To wrap itself around another cell, the engulfing cell must recruit new a membrane and completely reorganize its cytoskeleton, and these processes are regulated by the two pro-engulfment pathways, known as the CED-1 pathway (thought to be responsible primarily for membrane recruitment) and the CED-10 Rac pathway (thought to be responsible primarily for cytoskeletal rearrangement).

Cytoskeletal remodeling is also controlled by a host of other regulators outside these pathways, though, including a multi-talented protein called Abl kinase. It interacts with dozens of partners (creating effects that are mostly not understood in vivo), and its dysregulation is linked to at least two forms of cancer.

To explore the effects of Abl kinase in engulfment, the authors turned to the roundworm Caenorhabditis elegans, which produces an Abl kinase homolog called ABL-1. They found that disabling ABL-1 through mutation in animals defective for engulfment led to even more cells being engulfed, which is exactly what would be expected from the loss of an engulfment inhibitor. To quantify defects in the engulfment process, the authors simply counted corpses in the worm larvae, and they recognized that, rather than having a direct effect on engulfment itself, ABL-1 mutation might reduce corpse number by other means, such as suppressing cell death. But through direct observation of the developing larvae, they ruled out this and other alternatives, leading them to conclude that ABL-1 directly inhibited engulfment. The protein appears to exert its effect on the engulfing cell, because it inhibited engulfment when it was expressed everywhere except the engulfed cell.

**Figure:**
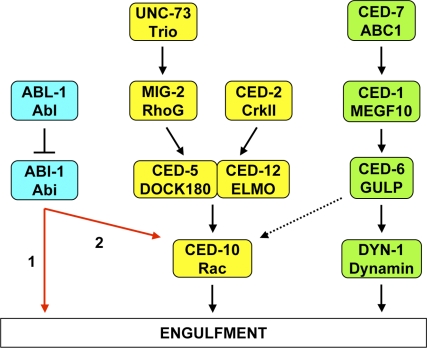
For many years, two molecular pathways were known to regulate the engulfment of apoptotic cells. Now, a third pathway has been identified in C. elegans that is inhibited by the Abl tyrosine kinase oncoprotein.

Mutation of *abl-1* was able to partially overcome the effect of complete inhibition of the membrane recruitment pathway but not the cytoskeletal rearrangement pathway. However, the cytoskeletal rearrangement pathway is also required for certain cell migrations, and mutation of *abl-1* did partially overcome the effect of complete inhibition of the that pathway on cell migrations. These data suggested that ABL-1 acts independently of both pathways, but the effect of its loss is simply too weak to overcome the engulfment defect of complete inhibition of the cytoskeletal regulation pathway. Thus, ABL-1 appeared to inhibit a third engulfment regulatory pathway.

The authors then explored the function of a known interactor with ABL-1, called ABI-1. In contrast to mutant ABL-1, mutations in ABI-1 reduced engulfment, just as mutations in the two previously known pro-engulfment pathways did. And it did so whether or not ABL-1 was mutated, suggesting that its effect is downstream of ABL-1.

The picture that emerges, then, is that ABL-1 acts directly on ABI-1 to inhibit it, and thus inhibits engulfment. Along with deepening the understanding of programmed cell death control, these results may have some direct therapeutic relevance. Engulfment of infected cells by macrophages is a key means for the body to maintain health, and engulfment of cancerous cells might also prevent cancer development. Abl kinase inhibitors might promote macrophage activity in these conditions. Impaired engulfment is a hallmark of systemic lupus erythematosus, suggesting that inhibiting Abl kinase might be of use here as well. Testing these ideas shouldn't take long—not only do such inhibitors exist, but at least two are already approved and marketed for other conditions.

